# Effects of Citrus Aurantium (Bitter Orange) on the Severity of First-Stage Labor Pain

**Published:** 2014

**Authors:** Masoumeh Namazi, Seddigheh Amir Ali Akbari, Faraz Mojab, Atefe Talebi, Hamid Alavi Majd, Sharareh Jannesari

**Affiliations:** a *Department of Midwifery, Faculty of Nursing and Midwifery, Shahid Beheshti University of Medical Sciences, Tehran, Iran, *; b *Department of Pharmacognosy, Shahid Beheshti University of Medical Sciences, Tehran, Iran, *; c *Department of Biostatistics, Faculty of Paramedicine, Shahid Beheshti University of Medical Sciences, Tehran, Iran. *

**Keywords:** *Citrus aurantium*, Pain, Complementary medicine, Medicinal plants, Aromatherapy, Labor

## Abstract

Considering that vaginal delivery is a painful process, the present study investigated the effects of *Citrus aurantium *on the severity of first-stage labor pain in primiparous women. This study was a randomized clinical trial conducted with 126 eligible primiparous patients. The pain severity of patients was measured at the time of enrolling in the study. In the intervention group, (aromatherapy) gauze squares were soaked in 4 ml of *C. aurantium *distillated water, and in the control group, gauze squares were soaked in 4 ml of normal saline; each gauze square was attached to the respective patients’ collar. The intervention was repeated every 30 min. Pain severity was measured after the intervention at 3–4, 5–7, and 8–10 cm cervix dilatations. The two groups were standardized with regard to age, profession, education, desire to conceive, and number and severity of uterine contractions. The Bishop’s score was also calculated. Before intervention, pain severity was the same for both groups, but following intervention, pain severity reduced in the intervention group at 3–4 centimeter (P < 0.05), 7–5 centimeter (P < 0.05), and 8–10 centimeter (P < 0.05) dilatations compared with that in the control group. The findings of the study revealed that aromatherapy using *C. aurantium *distillated water alleviates labor pain. This method is recommended because of its ease of use and low cost and because it is a non-aggressive method to reduce labor pain.

## Introduction

Pain is a common and integral part of childbirth (1). Contrary to progress in medical sciences, controlling labor pain is still a challenge in obstetrics (2). Tournaire states that delivery is a physiological process, and it is still associated with a severe, unendurable pain (3). Trout describes labor pain as a consequence of stimulation of neural receptors caused by uterine contractions that are passed to visceral, pelvic, and lumbosacral areas (4). Labor pain can contribute to mothers losing their psychological control and can be a key factor in traumatic delivery and mental disorders (5). 

Management and control of labor pain is a main objective of obstetric care and support (6). Attending to reducing the labor pain and paving the way for application of pain-reduction methods in hospitals and birth centers throughout the country will enhance mothers’ acceptance of natural labor (5, 7). Generally speaking, there are two methods in use for reducing the labor pain: pharmacological and non-pharmacological approaches (8). The pharmacological methods include systemic medication, general anesthesia, inhalational anesthesia, and topical anesthesia, while light therapy, aromatherapy, reflexology, hypnotism, massage, and acupuncture fall under the category of non-pharmacological methods (9). Simkin and Bolding believe that non-pharmacological methods should be preferred over the pharmacological ones for reasons of their lower cost, ease of application, non-invasiveness, the feeling of self-esteem it brings to women, and finally, patients’ participation (10).

One of the non-pharmacological methods of pain control is aromatherapy. Aromatherapy encompasses using aromatic herbal extracts and base oils (essential oils) for massage and other treatments (11). Different studies have shown that when used as inhalers, the oil essences create endorphins and reduce pain (12). A comprehensive study carried out in England on 8000 pregnant women during 1990–1998 showed the effects of aromatherapy on reduction of fear, pain, and anxiety associated with delivery. In this study, 61% women used lavender and frankincense to reduce fear and anxiety. Another 71% women identified rose flower extract as having a positive effect on pain reduction (10, 13). In a study by Vakilian *et al. *that investigated the effects of using lavender as an inhaler on labor pain results showed that the pain average for the lavender group was meaningfully lower than that of the control group (12). In a study that used peppermint oil as an inhaler, Ozgoli *et al*. showed it could reduce labor pain and anxiety in the first stage of delivery in primiparous women (14). Yip and Ying Tam’s study (2008) showed that *Citrus aurantium *was effective in reduction of moderate and severe knee pain (15). A study conducted between 2000 and 2002 by Mousely in England involved 80 pregnant women and showed that aromatherapy using lavender and frankincense extract had a positive effect on labor pain and anxiety reduction (16). A similar study in 2003 investigated the effects of aromatherapy on labor pain and showed that using jasmine, lavender and frankincense alleviated labor pain and reduced the need to take painkillers (17). 

*Citrus aurantium *oil is commonly used in aromatherapy. Bitter orange (*C. aurantium*) is derived from a small shrub that typically reaches to 3–4 meters in height and grows in the northern and southern areas of Iran. The flowers grow in isolation or in groups, dispersed on the branches. The flowers are scented with thick, juicy petals in yellowish white. On the surface of the leaves there are very small openings called stomata that can easily be seen; these can act as exit pathways for the plant’s essential oil (18, 19).

Only 0.2% of the density of *C. aurantium *flower is composed of its essential oil, called neroli oil. There are more than ten components in the *C. aurantium *oil, which are mostly the following monoterpens: limonene, linalool, linalyl acetate, geranyl acetate, geraniol, nerol, neryl acetate (20).

The oil has the effects of being an anti-depressive, anti-septic, anti-spasmodic, enhancer of sexual desire, and a mild sedative (2). The limonene found in the *C. aurantium *oil controls cyclooxygenase I and II, prevents prostaglandin activity and reduces pain (21). Although aromatherapy using other herbs has shown effects on the method on labor pain reduction, there is inadequate evidence based on clinical trials that have focused on the effects of *C. aurantium *on labor pain. Recognizing its sedative and pain-reducing effects the researchers focused on the effects of *C. aurantium *oil on labor pain reduction as an adjunct to midwives’ role in reducing labor pain and the increased interest in the use of pain-reduction methods with fewer side effects for mother and the fetus.

## Experimental

The present study was a randomized clinical trial and open label investigating the effects of *C. aurantium *on labor pain. The study was conducted with the permission of vice president for registrar affairs of Shahid Beheshti Medical University and approval of the University Ethics Committee. Written consent was acquired from the participants and the clinical trial was registered on the website of the Ministry of Health. The study was registered in the Iranian Center for Clinical Trials under registration No. N6 201301306807 IRCT. After consulting with statistical professor the minimum number of sample size calculated through the below formula: 

$n=\frac{{2(z_{\frac{\alpha }{2}}+z_{\beta }}^{2}\sigma^{2})}{{\left(\mu_{1}-\mu_{2}\right)}^{2}}=\frac{{2(1.96+0.48)}^{2}}{0.5^{2}}$

63, α=0.05, β=0.2

$\frac{\mu_{1}-\mu_{2}}{\sigma }=0.5:\mathrm{effect size}$

 A total of 126 pregnant women admitted to Valie-asr Hospital in Toyserkan (Hamadan Province, West of Iran) who were eligible to participate in the study were chosen through simple method of randomization. Sampling was carried out between June and September, 2013. The inclusion criteria were: Iranian ethnicity, being primiparous, aged between 18–35 years, full-term pregnancy, singleton pregnancy, cephalic presentation of the fetus, having automatic contractions, examining the dilatations (3–4 cm) at the moment of enrolling in the study, suitable pelvis status, having intact amniotic sac, not having consumed painkillers within 8 hours before enrolling in the study, absence of any identified liver, pancreas or respiratory diseases, not suffering from pregnancy and obstetric symptoms (*e.g.*, preeclampsia, chorioamnionitis, placenta abruption, abnormal fetal heart rate at the moment of enrolling in the study), absence of olfactory disorders or sensitivity to herbal medicine according to the participant. 

Participants were excluded from the study if urgent cesarean section was required before completion of the study, unbearable sensitivity to *C. aurantium *experienced, and the presence of signs indicating delivery, such as vaginal bleeding. The researchers chose the eligible participants from patients in the obstetrics ward of Valie-asr Hospital and provided them with necessary information. A written consent was obtained from all participants. The eligible primiparous women were then assigned on random days to either the *C. aurantium *or control groups. Randomization of the days was carried out using randomized table of numbers and on each day only aromatherapy or normal saline was used. The *C. aurantium *distillated water used in this study was produced by the Garreban Company, Iran. Each 100 mL of the product contained 8 mg *C. aurantium *oil, the density of which was measured and approved by the School of Pharmacy, Shahid Beheshti Medical University. In the aromatherapy group, gauze squares were soaked in 4 mL *C. aurantium *distillated water and in the control group gauzes were soaked in 4 mL of normal saline and were attached to the participants’ collars, with the intervention repeated every 30 min. The women’s pain severity was measured before and after intervention at dilatation stages of 3–4, 5–7, and 8–10 cm. Data analysis was carried out using the demographic and obstetrics questionnaire, observation and exam checklist, and the numerical scale of pain measurement. In order to examine the validity of the demographic and obstetrics questionnaire content validity method was implemented. In this way after reviewing related articles and reference books and according to the objectives of the research the demographic and obstetrics questionnaire perpetrated and approved by 10 members of Midwifery Faculty of Shahid Beheshti Medical University. In order to investigate the stability of the observation and examination checklist, parallel reliability was implemented. The observation and examination checklist completed by researcher and a colleague with experience matched with researcher for 10 Participants and the correlation coefficient was 0.85. The numerical pain scale has consistent correlation with other pain measurement scales (22) and has been used in other studies (14, 23-25). In a controlled randomized clinical trial in admitted patients in the first and second days after Cardiac surgery, the stability of the two pain rulers (*i.e*., visual and numerical) were measured using a 15-minute retest where the stability for the visual scale was between 0.73–0.82 and the numerical 10-digit score was 0.72–0.78 (26). Farrar *et al. *(27) carried out a retest in a 7–14-day interval to evaluate the reliability of the numerical pain scale and determined a correlation coefficient of 0.83. The pain measuring instrument is scaled from 0 to 10 with 0–3 indicating ‘mild pain’, 4–7 ‘moderate pain’ and 8–10 ‘severe pain’. The gathered data were analyzed using SPSS v22, independent T test, Chi-squared and Mann-Whitney U test. Parametric tests were performed where the data were normally distributed.

**Figure 1:**
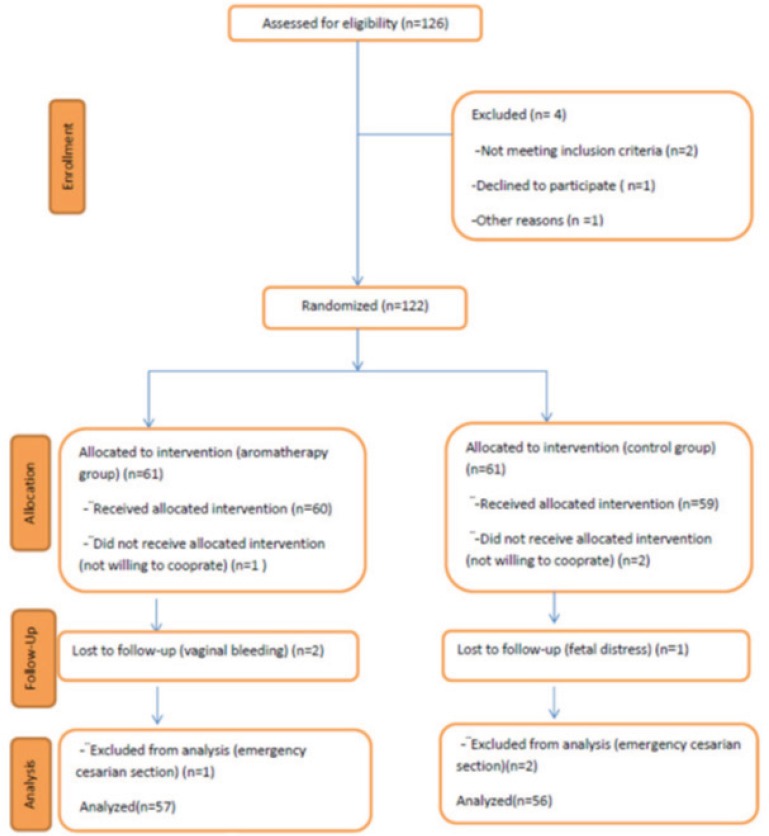
Consort flowchart of study. Figure 1. consort flowchart of study

## Results

The study was conducted on 126 primiparous women. Table 1 shows the personal, social and obstetrics characteristics of the participants. There was no meaningful difference between groups regarding participants’ age, education, profession, pregnancy age, receiving prenatal care, type of childbirth and the frequency and length of uterus contractions before the intervention (P>0.05).

The mean age of women in the aromatherapy group was 26.43 years (SD = 3.216) and in the control group it was 26.60 years (SD = 3.406). Most of the participants and their husbands in both groups had high school and lower level education and were housekeepers. The mean age of pregnancy in the aromatherapy group was 38.30 weeks (SD = 0.978) and in the control group was38.08weeks (SD = 1.067). Most of the participants in both groups had Wanted pregnancy. Most of the participants in both groups received prenatal care and also had normal vaginal delivery childbirth.

**Table 1:** Distribution of primiparous women based on their personal, social and obstetrics characteristics.

**Variable**	**Aromatherapy group**	**Control group**	**p-value**
Standard deviation and mean of mothers' age (year)	26.43±3.216	26.60±3.406	0.768
Education high school and lower university degree	79.4% 20.6%	82.5% 17.5%	0.967
Profession Housewife Working	79.4% 20.6%	82.5% 17.5%	0.650
Husband's Education high school and lower university degree	84.2% 15.8%	87.3% 12.7%	0.895
Husband's profession Unemployed Free work emploee	14.3% 69.8% 15.9%	12.7% 69.8% 17.5%	0.948
Standard deviation and mean of pregnancy age(week)	38.30±0.978	38.08±1.067	0.225
Wanted pregnancy	79.4%	82.5%	0.650
Receiving prenatal care Yes No	92.5% 4.8%	96.8% 3.2%	0.650
Type of childbirth Normal vaginal delivery Cesarian section	98.4% 1.6%	96.8% 3.2%	0.561
Standard deviation and mean of contraction length in 3-4 cm dilatation	44.08±0.703	43.94±0.759	0.275
Standard deviation and mean of contraction length in 5-7 cm dilatation	47.40±0.493	47.27±0.447	0.133
Standard deviation and mean of contraction length in8-10 cm dilatation	49.44±0.501	49.43±0.499	0.859
Standard deviation and mean of contraction frequency during 10 minutes in 3-4 cm dilatation	2.27±0.447	2.27±0.447	1.000
Standard deviation and mean of contraction frequency during 10 minutes in 5-7 cm dilatation	3.16±0.368	3.22±0.419	0.368
Standard deviation and mean of contraction frequency during 10 minutes in 8-10 cm dilatation	3.81±0.396	3.73±0.447	0.294

Before intervention, pain severity was the same for both groups, but following intervention, pain severity reduced in the intervention group at 3–4 centimeter (P < 0.05), 7–5 centimeter (P < 0.05), and 8–10 centimeter (P < 0.05) dilatations compared with that in the control group (Table 2).

**Table 2:** Distribution of average pain score of primparous women in different dilatations according to study groups

**Dilatation stages**	**The mean and standard deviation of the aromatherapy group**	**The mean and standard deviation of control group**	**Result of the independent t-test**
before intervention 3-4 cm dilatation 5-7 cm dilatation 8-10 cm dilatation	7.38±0.888 4.97±0.740 6.65±0.481 7.57±0.560	7.52±0.948 8.08±0.679 8.67±0.568 9.46±0.534	p=0.384 p<0.001 p<0.001 p<0.001

## Discussion

In the present study, the difference in pain scale of the two groups showed that aromatherapy using *C. aurantium *reduces participants’ labor pain. O’Flaherty *et al*. (2012) showed that aromatherapy using *C. aurantium *and lavender oils could be used to reduce the pain associated with burns (28). Since there were no other bodies of research focusing on the effects of *C. aurantium *on labor pain, previous studies using orange oil are mentioned here as it has a similar chemical composition (29). Rashidi Fakari *et al*. (2013) investigated the effects of orange oil on pain severity of the first stage of delivery in primiparous women. In this study of 150 primiparous women, pain severity was measured before and after aromatherapy. The results showed that aromatherapy using orange oil reduced pain in primiparous women (30). It is worth noting that in this study the pain severity was only measured at 3–4 cm dilatations, while in the present study, aromatherapy was continued until the end of the active phase and its effect on pain severity was measured. In a study in 2008, Yip *et al. *showed that massage and aromatherapy using a combination of orange and *Zingiber officinale *(ginger) was effective in reducing knee pain during the first week of treatment (15). Ozgoli *et al. *(2012) showed that using oral orange oil reduced breast pain caused by premenstrual syndrome (PMS) (31). The results of these studies are in line with the results of the present study. The aforementioned studies show the effects of aromatherapy on uterus-related pain like dysmenorrhea using orange oil, which has a similar chemical composition to those of *C. aurantium*. Because the cause of uterine pain in the present study and these other studies are the same, these results may be regarded as likely candidates to confirm the results of the present study. 

Aromatic oil used in aromatherapy reduces pain and instill tranquility by affecting the olfactory system through neurotransmitters in the olfactory glands and the limbic systems and motivating emotions (32–34). Prostaglandins cause pain and inflammation in the human body. These compositions are identified by cyclooxygenase I and II from arachidonic acid (35). Most of the painkiller and anti-inflammatory drugs reduce pain and inflammation by controlling these enzymes (21, 36). It seems that the limonene in *C. aurantium *oil controls the enzymes in prostaglandins and reduces pain (21). Limonene is one of the main components also found in fennel. The oil in these herbal medicines controls the contractions caused by oxytocin and prostaglandins and exert anti-uterine pain effects. Several studies have shown the positive effects of this herbal medicine in reducing dysmenorrhea (37–39). 

The researchers investigated all the participants regarding possible side effects. No serious side effects occurred during the study. The mean of the 1^st ^and 5^th^ minutes’ Apgar scores for infants born in both groups showed no significant difference (P>0.05). Aromatherapy using *C. aurantium *has shown no negative effects on the fetus. In this study, the research units were asked for their views on the degree of satisfaction for aromatherapy using *C. aurantium*. Responses indicated that 88.1% of participants in the aromatherapy group were satisfied with the method applied and 92.1% stated that they would use this method in future deliveries. 

Due to the limited number of studies on the effects of *C. aurantium*on labor pain and reports of no side effects, further studies on this strategy are strongly recommended to explore the pain-reduction biochemical mechanism of *C. aurantium*.

## Conclusion

The results of the study showed that aromatherapy using *C. aurantium *reduces labor pain. The method is recommended for implementation as an approach to reduce labor pain based on its low cost, ease of application, and non-invasiveness. 
